# Interplay Between TLR4 and Gelatinases in Tumour Growth and Metastasis

**DOI:** 10.3390/cells15090822

**Published:** 2026-04-30

**Authors:** Abdulfattah Al-Kadash, Peter Michael Moyle, Marie-Odile Parat

**Affiliations:** School of Pharmacy and Pharmaceutical Sciences, The University of Queensland, Brisbane, QLD 4102, Australia; p.moyle@uq.edu.au (P.M.M.); m.parat@uq.edu.au (M.-O.P.)

**Keywords:** Toll-like receptor 4, MMP, gelatinase

## Abstract

The modulation of the tumour microenvironment represents a pivotal step in tumorigenesis and metastasis and results from direct and paracrine cellular interactions. The innate immune Toll-like receptor 4 (TLR4) controls immune and inflammatory signalling in the tumour microenvironment. A growing body of evidence shows that TLR4 activation in cancer, immune and stromal cells upregulate gelatinase expression and activity, linking innate immune responses to extracellular matrix (ECM) remodelling. Gelatinases, or matrix metalloproteinases (MMP2) and (MMP9) play a pivotal role in tumour matrix degradation, thereby facilitating invasion, angiogenesis and metastasis. Interestingly, although TLR4 signalling in cancer cells and tumour-associated macrophages leads to different activation outputs, they can both induce gelatinases through NF-κB, MAPK, and Akt pathways. Evidence from clinical tumour tissues, co-culture models, in vivo and in vitro studies supports the crucial interplay between TLR4 signalling and gelatinases production in tumour growth and metastasis. An in-depth understanding of this crosstalk may reveal new therapeutic opportunities in targeted strategies.

## 1. Introduction

Tumours are characterised by invasive and metastatic properties associated with complex cell–cell and ligand-mediated interactions with the surrounding tumour microenvironment. These interactions facilitate tumour ability to evade the immune system, promote inflammation, change metabolic processes and restructure the surrounding tissue, which eventually facilitates tumour progression, invasion, angiogenesis and metastasis [1]. Within the tumour microenvironment, the extracellular matrix (ECM) is a fundamental supportive structure that consists of collagens, glycoproteins, proteoglycans and a wide range of secreted and entrapped molecules. The remodelling of the ECM is an essential step that enhances the invasive and metastatic phenotypes of tumour cells. This process is governed by different mediators and secreted enzymes, such as matrix metalloproteinases (MMPs) [2]. Gelatinases secreted by both cancer cells and other TME cells, including macrophages [3], endothelial cells [4], fibroblasts [5], and other immune cells cleave ECM proteins [6,7]. These MMPs are balanced by natural inhibitors known as tissue-inhibitors metalloproteinases (TIMPs) [8]. Increased production of MMPs is associated with tumour progression [9]. Additionally, MMP production is enhanced by the paracrine interaction between tumour cells and surrounding macrophages [10]. The production of MMPs is modulated by different receptors and signalling mechanisms, including the activation of toll-like receptor 4 (TLR4). While the role of TLR4 and the role of gelatinases (MMP2 and MMP9) in cancer has been abundantly examined, this review focuses on the interplay of these two pathways in cancer growth and metastasis. We searched PubMed for research articles containing in their title or abstracts free text synonymous terms for TLR4, MMP2 or MMP9, and cancer. We included full text articles published in English between January 2000 and July 2025. Furthermore, we searched the National Library of Medicine clinicaltrials.gov for up-to-date clinical trials related to our topic.

## 2. Toll-like Receptor 4

### 2.1. Toll-like Receptor 4: Biological Structure, Expression and Signalling Pathway

Toll-like receptors (TLRs) belong to the innate type 1 glycoprotein receptor family that recognises both internal stimuli, including damage-associated molecular patterns (DAMPs) released from tissue damage, and external stimuli, such as pathogen associated molecular pattern (PAMPs) released from different microorganisms. Ten types of TLRs have been identified in humans that are either cell membrane receptors binding external stimuli like proteins, lipids or LPS, (TLR1, TLR2, TLR4, TLR5, TLR6, TLR11) or intracellular, such as TLR3, TLR7, TLR8, TLR9, which are endosomal receptors [11].

TLRs are expressed at different levels among cancer cells with different observational outcomes, sometimes correlating their levels of expression to survival rate. For instance, high expression levels of TLR9 in oesophageal cancer is associated with high tumour proliferation [12]. Moreover, both TLR7 and TLR8 are reported to be highly expressed in pancreatic cancer, and TLR4 level was significantly elevated in lung cancer and correlated with poor overall survival rate [13,14]. This indicates the crucial role of TLRs in the cancer context and their potential as therapeutic targets.

**Figure 1:**
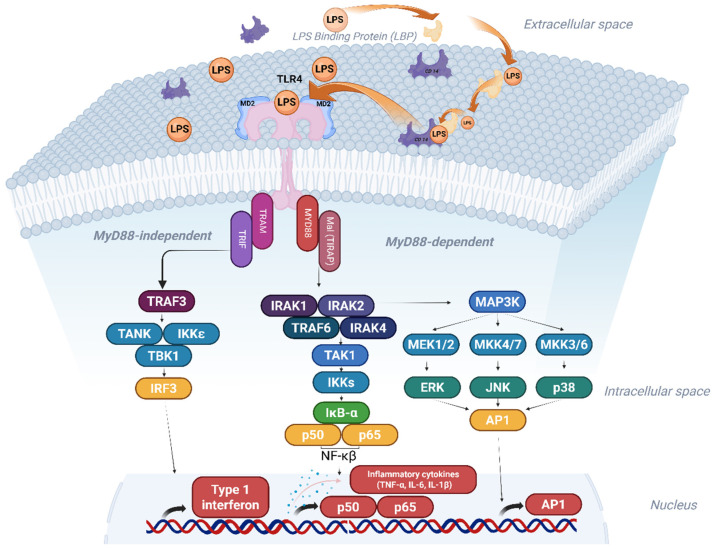
TLR4 signalling pathways. LPS-binding protein (LBP) binds LPS and brings it to CD14. CD14 transfers LPS to TLR4 via co-receptor MD2. The binding of LPS to TLR4 triggers TLR4 homodimerisation and initiates two different signalling pathways; MyD88-depdendent pathway, which activates NF-κB and induces proinflammatory cytokines and chemokines, and the MyD88-indepdent pathway, which occurs after endocytosis of TLR4 and results in the induction of type 1 interferon. This figure is adapted from [17]. Created in BioRender (Al-Kadash, A. 2026 https://BioRender.com/te5wu4o).

Activation of TLRs elicits downstream signalling. In the case of TLR4, the prototypical activating ligand is the PAMP LPS. A serum protein called LPS binding protein (LBP) binds LPS and brings it to CD14, which can be soluble or bound to the cell membrane. CD14 delivers LPS to TLR4 via its co-receptor MD2. Homodimerisation is induced when LPS attaches to TLR4/MD2. This initiates two different pathways depending on the set of adapter proteins that are recruited (Figure 1). The first pathway is activated by the downstream adaptors Toll/interleukin-1-receptor (TIR)-domain-containing adaptor protein (TIRAP) and myeloid differentiation primary-response protein 88 (MyD88), and results in NF-κB activation and the induction of proinflammatory genes, immune mediators and chemokines, such as TNF-α, IL-6 and IL-1β. These cytokines are part of the early inflammatory response and act locally and systemically to trigger immune cells and recruit them to the infection site. In addition, chemokines act as signalling proteins to guide immune cells to the infection or the inflammatory sites. A second activation pathway is mediated by TIR-domain containing interferon-β (TRIF) coupled with the TRIF-related adapter molecule (TRAM), which occurs from the endosomes after endocytosis of TLR4 and results in the induction of type I interferons [15,16].

### 2.2. Toll-like Receptor 4: Expression and Function in Macrophages

TLR4 is abundantly expressed by monocytes, macrophages and granulocytes but few to undetectable levels are present in dendritic cells. Other cell types or tissues with elevated TLR4 expression levels include adipocytes and spleen, followed by dermal microvessel endothelium, brain endothelial cells, brain-microglia, small intestine, colon, ileum, lung, ovary, and placenta. In contrast, minimum TLR4 expression were detected in the kidney, liver, and brain [18].

TLR4 is one of several receptors enabling macrophage polarisation, maturation and intracellular signalling. The activation of TLR4 in macrophages drives the expression of genes that are considered M1 hallmarks (e.g., TNF-α, IL-6, IL-8, IL-1β, iNOS). In fact, when eliciting classical (M1) activation of macrophages in vitro, the typical experimental protocol employs a mixture of LPS and IFN-γ [19]. Furthermore, TLR4 expression is heightened in M1 macrophages and TLR4 has been referred to as a M1 marker, although TLR4 expression is not unique to M1 [20,21]. Conversely, TLR4 deficiency promotes M2 polarisation [22].

M1 macrophages play essential roles in host defence and protection. However, M1 macrophages can be implicated in chronic inflammatory responses. For instance, M1 macrophages stimulated by endogenous DAMPs that activate TLR4 can produce proinflammatory cytokines and exacerbate renal injury [23]. In the context of cancer, the balance of M1 and M2 in the TME is crucial [24]. In early stages of tumour growth, cancer cells have a weak ability to reprogram macrophages, and they express ligands or receptors that can be recognised by macrophages for phagocytosis and elimination. In addition, tumour cells in the early stages invade and disrupt surrounding tissues, producing inflammatory responses that recruit and polarise M1 macrophages. These macrophages can exhibit antitumour effects and secrete chemokines (e.g., CCL5, CSCL10) that can recruit Th-1 cells [25]. The expression of TLR4 is central in the activation and polarisation of macrophages.

### 2.3. Toll-like Receptor 4 in Cancer

The role of TLR4 in cancer is ambiguous. On the one hand, TLR4 expression and/or activation on cancer cells is generally (but not always) associated with increased aggressiveness. On the other hand, activation of TLR4 on immune cells is reported to be protective in the context of tumours. Several studies indicate that the activation or the increased expression levels of TLR4 are associated with worst prognosis. Specifically, TLR4 has been shown to be overexpressed in tumours compared to normal tissues [26,27]; TLR4 activation and expression enhance tumour escape from the immune system [28,29], increase chemoresistance in bladder cancer [30], enhance migration and invasion of colorectal cancer [31], while TLR4 gene disruption was protective towards radiation-induced lymphoma [32]. In contrast, other studies indicate lower TLR4 expression in tumour tissue compared to healthy tissue [33,34], or TLR4 expression to be associated with improved antitumour immunity [26] and better prognosis [30,35]. This marks the importance of further investigating the role of TLR4 signalling and its interaction effects with immune system in organ-specific study. Table 1 summarises the effect of TLR4 activation on cancers in different organs.

Many studies employ LPS as a pharmacological tool to activate TLR4 in experimental models. While in real-life tumours, microbial products from local microbiota can infiltrate tumours, DAMPs are more relevant. DAMPs include a wide range of mediators, such as HMGB1, S100, heat-shock proteins, uric acid, calreticulin, annexin A1, and adenosine that reside inside the healthy cells [99,100,101,102,103,104,105]. Upon cell death or stress, these molecules are released and activate receptors expressed by different cell types, including macrophages. DAMP release from necrotic cells is induced by chemotherapy and radiation [106,107,108,109,110]. DAMPs are also produced from the ECM [111,112,113,114].

HMGB1 is a DAMP that has been extensively studied for its dual role in enhancing or antagonising tumourigenesis. On the one hand, HMGB1 sustains a pro-inflammatory environment, promotes immunosuppression, invasion, metastasis and angiogenesis; on the other hand, it increases autophagy, modulates genomic instability, interacts with tumour suppressor genes and inhibits cancer cell aerobic respiration to provide anti-tumour effects [109,115,116,117]. In a seminal study, HMGB1 released by dying tumour cells upon radiation or chemotherapy was shown to activate TLR4 on dendritic cells and tumour antigen-specific T-cell immunity [118]. TLR4 expression by the dendritic cells was required for the response against dying tumour cells in vivo, and breast cancer patients who carry a TLR4 loss-of-function allele relapse more quickly after radio- and chemotherapy than those carrying the normal TLR4 allele [118]. These data clearly demonstrate the importance of immune cell TLR4 expression and activation in anti-cancer immunity.

Accordingly, several recently completed clinical trials evaluated TLR4 as a therapeutic target in different types of cancers (Table 2). Although the results are in several cases yet to be finalised, the evidence for a clinical benefit does not, at this point in time, seem compelling.

## 3. Matrix Metalloproteinases

### 3.1. Matrix Metalloproteinases: Types and Functions

The ECM provides a structural supportive network for all tissues, regulating signalling and cell morphology. It is composed of collagens, glycoproteins, laminins, proteoglycans, and elastin. The ECM undergoes dynamic remodelling by proteolytic enzymes, primarily matrix metalloproteinases enzymes (MMPs), a family of zinc-dependent enzymes that contain calcium, and are reported to promote cancer progression through degradation of the ECM [124]. The MMPs include 28 types that have been identified in vertebrates, including 24 expressed in humans. The MMPs are mostly secreted in the extracellular environment or bound to cell surface, but have also been reported inside the nucleus, cytosol, and the mitochondria [124,125,126,127].

MMPs can be classified into (i) archetypal MMPs; including collagenases (MMP1, MMP8, MMP13), stromelysins (MMP3, MMP10, MMP11), and others (MMP12, MMP19, MMP20, MMP27), (ii) gelatinases (MMP2, MMP9), (iii) furin-activated MMPs, including secreted (MMP21, MMP28), type I transmembrane (MMP14, MMP15, MMP16, MMP24), type II transmembrane (MMP23A, MMP23B), and GPI-anchored (MMP17, MMP25) [128,129].

**Figure 2:**
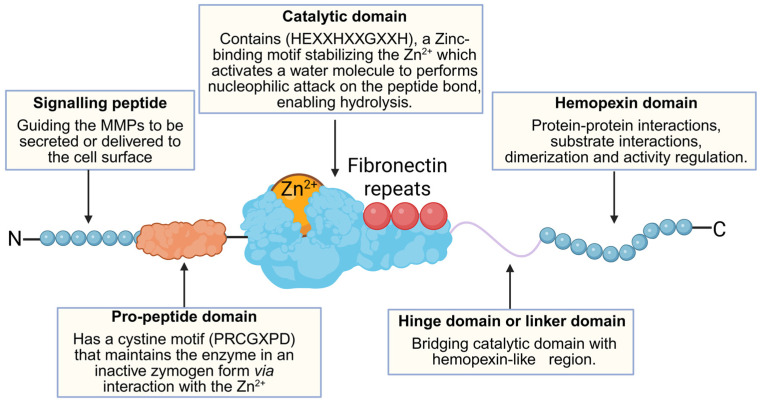
MMP structural domain functions shown on the example of gelatinases. Created in BioRender. Al-Kadash, A. (2026) https://BioRender.com/qy0q926.

All MMPs share a similar structure with a signalling peptide at the N-terminal region, followed by a pro-peptide domain, then a catalytic domain, a linker region, and a hemopexin domain, which constitutes the C-terminus except for membrane-bound MMPs (Figure 2) [130].

After their synthesis in the endoplasmic reticulum, MMPs are transferred for post translational modification in the Golgi apparatus, and kept in their latent inactive form known as pro-MMP [131]. The activation of MMPs can happen either intracellularly or extracellularly. After removal of the signal peptide, the cystine-Zn^2+^ is cleaved and the pro-peptide removed, forming the active MMP. MMP activation is enhanced by low pH, reactive oxygen species, and increase in temperature. After activation, MMPs initiate degradation of the ECM through proteolysis of collagens, glycoproteins, or other MMPs. Through their ECM remodelling, MMPs are essential in healing and organ development [130,132]. In addition, MMPs are highly upregulated during diseases and have been studied in detail in the context of tumourigenesis.

### 3.2. Matrix Metalloproteinases MMP2 and MMP9 in Cancer

MMP2 and MMP9 are also termed gelatinases. MMP2, or gelatinase A, has a molecular weight of ~72 kDa, while MMP9, or gelatinase B, has a molecular mass of ~92 kDa. Both have a catalytic activity that is central in tumour invasion, metastasis and angiogenesis, as in addition to gelatin they degrade basement-membrane collagens especially type IV collagen. Specifically, MMP2 cleaves type I, IV, V, VII and X collagens and laminin, while MMP9 degrades types III, IV, and V collagens, aggrecan, fibronectin and non-matrix proteins, including chemokines and cell-surface proteins, thereby releasing cell-regulating activities [133].

Both MMP2 and MMP9 secretion can be triggered by cytokines, tumour cells signalling, or inflammation [134]. Furthermore they are subject to post-translational modifications, including N-linked glycosylation, phosphorylation at multiple residues [135]. In the TME, these modifications can change secretion, stability, activation, and pericellular localisation of MMP2 and MMP9, which in turn modulates invasion and metastasis. MMPs enhance tumour aggressiveness in different ways. For instance, in breast cancer, MMP2 enhances cells motility, while the inhibition of MMP9 decreases breast cancer cells invasion [136]. In addition, co-culture of macrophages with breast cancer cells upregulates MMP9 secretion by a TNF-α-mediated mechanism and promotes invasion [137]. Gelatinase-neutralising antibodies decrease early lung foci [138,139]. In vivo preclinical studies corroborate the protective roles of gelatinase inhibition in cancer; for instance, decreased microvessel density and substantial decrease in VEGF availability was evidenced after MMP9 suppression [140]. Reviews support the correlation of MMP2 with worse breast cancer prognosis [141,142]. Similar correlations with poor prognosis were noted with MMP9 expression in breast cancer [143,144]. In lung cancer, MMP9 elevated expression is associated with poor survival in NSCLC [145]. Lastly, MMP9 offers diagnostic value in discriminating between hepatocellular carcinoma and hepatitis C Virus-related cirrhosis [146].

This made targeting MMP2 and MMP9 an attractive tumour treatment strategy. A variety of approaches to inhibit MMPs were tested, including direct gelatinase inhibitors with broad MMPs inhibition spectrum (e.g., prinomastat, marimastat, and MMP9-selective inhibitors (e.g., JNJ-0966 [147,148]). Further inhibitors that were evaluated for anti-gelatinase activity include phytochemicals, such as sanguiarine [149], or hinokiflavone [150]. In addition, other strategies were designed to indirectly inhibit MMP production by targeting of the pathways involved in their expression, such as MAPK/AP-1 and NF-κB, or AKT/IKK/NF-κB, since NF-κB is an essential modulator of MMP production [151,152,153,154]. Collectively, these inhibitors not only decrease MMPs production but also suppress cancer aggressiveness and its hallmarks. These studies demonstrate the significant role of MMPs in the therapeutic context for cancer treatment, and the importance of understanding the exact biological pathways that affects their expression.

We therefore analysed the literature on gelatinases in cancer as a function of the organ where the primary tumour originates. Table 3 summarises the role of gelatinases in various types of cancers. Both MMP2 and MMP9 tend to promote cancer progression and enable proliferation, invasion, and metastasis regardless the origin of the tumour. However, contradictory findings are also reported in the literature indicating that in some models, MMP2 and MMP9 inhibit the progression of cancer. These discrepant findings might rise from the differences in the experimental design, sample size, method of detection of MMPs, treatments used, cell line differences, TME, and stage of cancer.

As appears from Table 3, the majority of studies indicate an implication of gelatinases in the aggressive phenotype regardless of the organ of origin of the cancer. Across a wide range of malignancies, the data show that dysregulation of MMP9, and to a lesser extent MMP2, is a feature of cancer aggressiveness with pronounced association with cancer cell invasion, metastasis and clinical features, such as tumour stage, lymph-node involvement, poor prognosis, resistance to treatment or reduced survival. Moreover, functional studies show that MMP9 pharmacological or genetic inhibition can suppress cell proliferation, migration, angiogenesis, invasion and metastatic burden in vitro and in vivo. On the other hand, MMP2 exhibits more context-dependent alterations and may act as a prognostic biomarker in certain cancers.

Because pharmacological or molecular downregulation of MMPs emerges from preclinical studies as an efficient therapeutic approach, a lot of focus has been placed on developing and testing MMP inhibitors as an anticancer therapeutic strategy starting in the 1980s. Remarkably, despite highly promising anticancer effects in vitro and in animal models, no clinical benefits were achieved in clinical trials of cancer treatment [266,267,268] with additional obstacles related to oral bioavailability, fast elimination, lack of selectivity, variable responses and toxicity [269]. On the other hand, the US Food and Drug Administration approved the tetracycline antibiotic doxycycline as the only collagenase inhibitor indicated for periodontal diseases [270]. Accordingly, despite promising results of MMP inhibitors in vitro, this therapeutic approach lacks effectiveness in clinical studies [271,272,273]. It has been suggested that rather than administering an inhibitor, preventing the induction of gelatinases in the tumour microenvironment would be a better approach.

## 4. Toll-like Receptor 4 and Gelatinases Crosstalk in Cancer

### 4.1. Activation of TLR4 Upregulates MMP9 in Macrophages

The activation of TLR4 in THP-1 cells via exogenous and endogenous ligands upregulates MMP9 production in a dose-dependent fashion. LPS activation of THP-1 TLR4 causes decreased cell proliferation, increased production of MMP9 and macrophage M1 polarisation. Furthermore, LPS stimulation enhanced the upregulation of TLR4 [274]. In another study, LPS caused partial decrease in MMP9 mRNA three hours post-treatment, followed by upregulation at 6 and 12 h. Furthermore, an increase in MyD88 protein (which is essential for TLR4 recognition and signalling via LPS), as well as IκB-α degradation, p65 translocation to the nucleus and p38 (p-p38) and ERK1/2 (p-ERK1/2) phosphorylation, were detected [275].

Additionally, endogenously released DAMPs activate TLR4. Clusterin is a glycoprotein expressed at low levels under homeostasis and higher levels under inflammatory conditions, such as ischaemia and cancer, and works as a TLR4 ligand [276,277]. Clusterin induces upregulation of MMP9 production without affecting MMP2 in human primary monocytes, THP-1, peritoneal mouse macrophages, and RAW264.7. The LPS sequestering agent polymyxin B suppressed LPS-induced but not clusterin-induced upregulation of MMP9, eliminating the possibility of LPS contamination of the clusterin. Clusterin upregulates MMP9 via ERK1/2 signalling and PI3K/Akt/NF-κB signalling cascades [278].

Notably, lipid rafts are essential for TLR4 activity [279], as the disruption of cholesterol-enriched plasma membrane surrounding TLR4 significantly reduces activation of TLR4, thus decreasing MMP9 production after clusterin stimulation of Raw 264.7 cells [280]. Additionally, TLR4 activation upregulates MMP9 levels in macrophage-like cells generated by PMA-stimulation of human monocyte U37 cells [281]. These studies demonstrate the upregulation of MMP9 in macrophage, macrophage-like, and simulated macrophage cells via TLR4-activated signalling cascades.

### 4.2. Effect of TLR4 on MMP9 in Cancer Cells

#### 4.2.1. Pancreatic Cancer

TLR4 was found to be positively correlated to pancreatic cancer tumour size and aggressiveness [282]. Palmitic acid (PA) treatment activates TLR4 in AsPC-1 cells and induces the secretion of reactive oxygen species (ROS) as intermediate molecules, which in turn induce NF-κB activation and promote MMP9 transcription and secretion. In addition, the activation of TLR4 enhanced AsPC-1 cells invasion, which was suppressed by either a TLR4 inhibitor or MMP inhibitor (GM6001). The increase in MMP9 was significantly inhibited by either a TLR4 inhibitor, or ROS inhibitor, or NF-κB inhibitor. Aligning with other studies that support the role of TLR4-mediated NF-κB activation in chemoresistance and cancer proliferation [283], this study shows TLR4-NF-κB activation enhances MMP9 production, which ultimately promotes cancer invasion [284].

In another study, the in vitro treatment of pancreatic cells with LPS enhanced cell invasion without affecting cell proliferation. LPS significantly upregulated MMP9 mRNA and induced NF-κB activation. The transfection of pancreatic cells with MMP9 siRNA, NF-κB decoy, TLR4 siRNA, MyD88 siRNA all inhibited LPS-induced invasion. Furthermore, IκB-α-mutant cells had reduced invasion. This shows that the activation of TLR4/Iκβ-α/NF-κB/MMP9 is responsible for cell invasion in pancreatic cancer [285].

#### 4.2.2. Breast Cancer

Accumulating evidence shows that TLR4 activation also plays a crucial role in breast cancer [48]. The expression of TLR4 is detected in breast cancer cell lines MDA-MB-231 and MCF-7 in the cytosol and membrane. Linoleic acid enhances breast cancer cell migration and invasion while a TLR4 inhibitor completely suppresses the induced migration and partially inhibits invasion. Furthermore, linoleic acid increased MMP2 protein levels up to 36 h post treatment and MMP9 protein levels up to 48 h post treatment. The production of MMP9 is suppressed by TLR4 inhibition. A proposed mechanistic explanation for linoleic acid-induced TLR4 activation was activation of FFAR 1 and FFAR4 receptors, leading to secretion of molecules that transactivate TLR4 in breast cancer cells but not mammary normal epithelial cells. However, further studies are needed to confirm this assumption [51].

Another study confirms that treatment of breast cancer cells with LPS upregulates TLR4 and MyD88 protein expression, with increased MMP2, MMP9 and VEGF secretion in a dose-dependent response. The activation of TLR4 enhanced breast cancer cell invasion and metastasis in vitro. Furthermore, breast cancer cells transplanted in female nude mice led to higher tumour weight, increased lymph node status, and enhanced metastatic lesions in the liver if the cells were pretreated with LPS prior to inoculation [286].

The mechanism by which TLR4 activation induces MMP9 transcription in breast cancer cells involves a kinase named T-LAK cell-originated protein kinase (TOPK). LPS induced TOPK phosphorylation, upregulated TLR4 receptor expression, enhanced MMP9 and VEGF production in MCF7 cells. TOPK siRNA and pharmacological inhibition experiments demonstrated that TOPK is essential for NF-κB-mediated MMP9 induction by LPS and eventually cell invasion and migration [287].

The importance of TLR4, its endogenous ligand HMGB1 and MMPs in breast cancer has been highlighted by genetic polymorphism studies. In a sample of more than two thousand Korean women, single nucleotide polymorphism (SNP) in MMP2, TLR4 and HMGB1 were associated with disease-free survival and/or overall survival [288]. Specifically, two SNPs of *MMP2* gene (rs243867 and rs243842) demonstrated statistically significant associations with a poor breast cancer disease free survival, while rs4145277 in the *HMGB1* gene and rs243842 in the *MMP2* gene were significantly associated with a poor overall survival. In contrast, rs7045953 in *TLR4* was significantly associated with a good overall survival.

#### 4.2.3. Lung Cancer

In lung cancer, HMGB1 expression was found to be a determinant of invasion and metastasis, with HMGB1 expression in non-small lung cancer specimens significantly associated with advanced stage and MMP9 expression. Experimental downregulation of HMGB1 in lung cancer cell lines in vitro reduced MMP9 expression and metastatic features in vitro, confirming a functionally important connection between HMGB1 and MMP9 in lung cancer [289]. HMGB1 can be released from both immune cells and necrotic cells and binds to both RAGE and TLR4, initiating down signalling cascades aggravating inflammatory status and increasing proinflammatory cytokines, including IL-6 and TNF-α [290,291]. In vitro, exogenous HMGB1 significantly enhances invasion, MMP2, and MMP9 in lung cancer cells, whereas blocking MyD88 significantly inhibited this HMGB1induction of MMPs [292].

#### 4.2.4. Prostate Cancer

In prostate cancer, TLR4 expression was shown to be increased in cancer compared to normal tissue [293]. Furthermore, siRNA knockdown of either TLR4 or COX2 inhibits cell proliferation, migration and invasion with significant downregulation of both MMP2 and MMP9 in prostate cancer cells, and upregulation of the MMP inhibitor TIMP-1. This study indicated that COX2 might be involved in the pro-invasive effect of TLR4 [293].

#### 4.2.5. Ovarian Cancer

The expression of TLR4 was shown to differ among ovarian cancer cell lines, which was reflected by the consequences of TLR4 activation. Specifically, higher TLR4 mRNA in SK-OV-3 ovarian cancer cells was associated with a more pronounced mesenchymal phenotype, cell migration and invasion upon LPS treatment compared to Caov-3 ovarian cancer cells. LPS upregulated MMP2 and MMP9 protein levels in SK-OV-3 cells in vitro. This particular study further expanded on PI3K as a mediator of TLR-induced cell invasion and EMT [294].

#### 4.2.6. Liver Cancer

TLR4 mRNA was shown to be elevated in hepatocellular carcinoma (HCC) tissue compared to normal liver tissue, with overall survival significantly lower in TLR4 high group compared to TLR4 low group [295]. Higher TLR4 was also noted in HCC cell lines compared to normal liver cells in vitro. In this study, TLR4 downregulation mediated the protective effect of miR-122 on liver cancer, which correlated with reduced MMP9 protein [295]. An in vitro study showed that LPS dose-dependently decreased MMP2 activity in gelatin zymography in HepG2 cells. On the other hand, LPS upregulated MMP2 and TIMP-2 gene expression and downregulated TIMP-1 gene expression. The authors suggested that the apparent decreased MMP2 gelatinolytic activity might be due to the increased levels of MMP-2/TIMP-2 complex in conditioned media, leading to the decreased motility and invasiveness of HepG2 cells [296].

#### 4.2.7. Melanoma

While many studies show MMP induction via TLR4 signalling, others show that TLR4-independent pathways also exist in cancer cells. For instance, in inflammatory sites, small hyaluronic acid (sHA) fragments produced from the ECM can activate TLR4. In vitro treatment of sHA upregulates MMP2 mRNA in melanoma cells with increased p65 nuclear translocation. While the MMP2 active form was detectable only on the treated melanoma cells, the pro-form was significantly increased. However, the knockdown of TLR4 did not affect sHA-induced upregulation of MMP2 mRNA [297].

#### 4.2.8. Colorectal Cancer

LPS was shown to upregulate MMP2 and MMP9 protein levels in HT-29 colorectal cancer cells whereas it only significantly increased MMP2 protein levels in SW620 cells [298]. TLR4 expression was increased in both cell lines by LPS treatment, as were invasion and migration.

#### 4.2.9. Glioblastoma

Both glioblastoma cell lines and tissue express abundant TLR4 and MMP9. The effect of LPS was tested on two glioblastoma cell lines. LPS increased proliferation, but not migration, in both cell lines. Following LPS activation of TLR4 pathway, MMP9 active form levels increased in U87 supernatant but decreased in U118 glioblastoma cell line supernatant compared with their controls [299].

In summary, in addition to the previous studies in Table 1 and Table 3 showing individual correlations between TLR4 activation or MMP9 production and increased tumour invasiveness, these studies demonstrate that the activation of TLR4 in cancer cells by endogenous ligands, such as HMGB1, exogenous ligands, such as LPS, or through transactivation by other receptors, can lead to increased MMP9 production or expression. Several mechanistic mediators, such as ROS, PI3K signalling, TOPK, COX-2, or NF-κB signalling, have been proposed. Overall, the TLR4-NF-κB-MMP9 axis is predominantly associated with enhanced tumour aggressiveness features but variability observed across tumour types underscores that this pathway may be context-dependent. Nonetheless, inhibition of this axis provides interesting strategies to target tumour growth and metastasis.

### 4.3. Macrophage-Cancer Cells Co-Culture Results in Upregulation of MMP9

Several studies indicate that TLR4 may mediate MMP production in co-culture systems with macrophages and cancer cell lines. In vitro co-culture with M2-polarised TAMs increased pancreatic cancer cells proliferation, migration, and pro-invasive features, including MMP2 and MMP9 proteolytic activity [300]. Interestingly, co-culture also upregulated TLR4. The knockdown of TLR4, or TLR4-neutralising antibodies inhibited co-culture-induced upregulation of TLR4, MMP2, MMP9, EMT markers and decreased tumour cell aggressiveness induced by the co-culture [300]. This indicates that the upregulation of MMP2 and MMP9 in macrophage-cancer cell co-cultures is mediated by TLR4 signalling. In a similarly designed study employing ovarian cancer cells, co-culture with polarised M2-macrophages enhanced cell proliferation, invasion and migration. The co-culture upregulated TLR4 and HMGB1 mRNA and protein levels as well as MMP9, and HMGB1 knockdown in the macrophages significantly decreased TLR4 expression and MMP9 production, with decreased co-culture-induced proliferation, invasion and migration [301]. Although correlative in this study, the relationship between TLR4 and MMP9 may play a role in the TME. A study employing co-culture of macrophages and oral squamous cell carcinoma cells showed that HMGB1 knockdown inhibits co-culture-induced proliferation, invasion and migration. Interestingly, the HMGB1 knockdown downregulated TLR4 but not RAGE in macrophages. The dampening of HMGB1 in macrophages significantly decreased PD-L1 and MMP9 protein levels [302]. While this study did not link MMP9 production to TLR4 in macrophage-cancer cell co-culture, it indicates that HMGB1 contributes to the TLR4 expression and MMP9 in parallel.

Co-culture studies allow the paracrine interaction between different cell types of the TME while allowing to dissect out the cross talk—in this case the insight that gelatinases are secreted by both macrophages and cancer cells, the importance of M2 polarised TAMs, and the role of upregulation of TLR4 expression and TLR4 activation. Positioned between single cell culture and in vivo experimentation, these studies also point to the benefits of inhibiting TLR4-mediated increase in gelatinase activity.

### 4.4. Toll-like Receptor 4-MMPs in In Vivo Studies

MMP9 induction mediated by TLR4 activation may be involved in the peripheral neuropathy caused by chemotherapy in vivo. Paclitaxel is a commonly used drug to treat breast cancer, and it is associated with peripheral neuropathy [303]. The administration of paclitaxel induced TLR4 activation and significantly upregulated TLR4, p-p38 and NF-κB protein expression in vivo together with significant increase in MMP9 levels in the immunostained sciatic nerve after paclitaxel treatment. Interestingly, modulating paclitaxel-induced TLR4 activation and the induction of MMP9 without affecting paclitaxel antitumour effects reduced paclitaxel-induced peripheral neuropathy [304]. HMGB1-induced MMP9 contributes to the cisplatin-induced cognitive impairment, which is prevented in MMP9 knockout mice or by HMGB1 inhibitor pretreatment [305]. Other studies support the findings that the inhibition of TLR4 contributes to enhanced chemotherapeutic efficacy [88].

What are the clinical implications of TLR4-mediated increase in gelatinase action? Activation of the TLR4–MMP9 axis is consistently associated with enhanced extracellular matrix remodelling, tumour invasion, metastatic potential, and inflammatory crosstalk within the TME, while its inhibition attenuates these aggressive features. In vivo, modulation of TLR4-mediated MMP9 induction can reduce chemotherapy-associated adverse effects. Although these findings are derived from preclinical systems, they suggest that TLR4 and MMP9 may represent informative biomarkers of tumour aggressiveness and potential therapeutic targets for limiting invasion, metastasis, and treatment-related toxicity, warranting further validation in clinical settings.

## 5. Conclusions

TLR4 activation upregulates MMP2 and MMP9 expression and activity in cancer cells, macrophages, endothelial and stromal cells, promoting matrix remodelling, invasion, neovascularisation and intravasation. Matrix degradation can in turn release ECM protein fragments or DAMPs that further inflammation and proteolysis. In addition to these protumour effects, TLR4 engagement also results in beneficial effects via anti-tumour immunity. As a consequence, TLR4 activity modulation for therapeutic purposes may result in mixed effects. Strategies combining TRL4 modulation and gelatinase inhibition as part of a multitargeted approach are worth evaluating. A better understanding of the ligand and cell-type specificity of TLR4-MMP crosstalk may allow development of more selective modulators.

## Figures and Tables

**Table 1:** TLR4 expression and associated effects in tumours from different organs.

Type of Cancer	TLR4 Expression Variations and/or TLR4-Mediated Effect
Bladder cancer	TLR4 expression is higher in bladder cancer cell lines compared to normal cells, and in bladder cancer tissues compared to normal tissues. The activation of TLR4 promotes cancer progression in vitro, and its pharmacological inhibition reduces tumour size and weight in vivo [27]. Lower TLR4 mRNA expression levels in tumour compared to healthy tissue [33]. TLR4 expression is decreased in tumour compared to normal tissue, and lower TLR4 expression correlates with aggressiveness and poorer survival [30]. Low expression of TLR4 in tumours was associated with poor prognosis. siRNA knockdown of TLR4 in bladder cancer cells decreased proliferation but increased migration and invasion [36]. TLR4 activation in bladder cancer cell line increases PD-L1 expression [28].
Colorectal cancer	TLR4 activation did not change TLR4 expression in colon cancer cells, or their proliferation significantly, but it induced resistance to TRAIL-induced apoptosis [29]. TLR4 activation induces resistance to CAR-T cell therapy both in vitro and in vivo [37]. IHC of human tissues shows higher TLR4 expression and higher combined TLR4/MyD88 expression in colorectal carcinoma compared to adenoma tissue. High TLR4 expression also associated with perineural invasion and higher tumour budding [38]. Downregulation of TLR4, MyD88 via the phytochemical gastrodin is associated with decreased colitis and colorectal cancer phenotypes in vivo [39]. CD14+-sorted primary colorectal cancer treated by LPS showed enhanced cell migration and proliferation [31]. TLR4 activation enhances colorectal cancer cell proliferation [40]. TLR4 inhibition partially reduces tumour size, and LPS trap inhibits liver metastasis in orthotopic colorectal tumour in vivo [41].
Brain cancer	TLR4 inhibition suppresses cell invasion, migration and MMP2 production in glioblastoma cells in vitro and decreases tumour growth rate in vivo [42]. Tissue mRNA analysis shows higher TLR4 expression in glioblastoma patients with leptomeningeal disease compared with glioblastoma patients without leptomeningeal disease. In addition, TLR4 inhibition enhances survival rate in murine model [43]. TLR4 activation is implicated in immune escape and radioresistance induced by the transmembrane receptor TREM 2, which interacts with HMGB1 and activates TLR4/Akt signalling [44]. HMGB1 released from glioblastoma cells activates TLR4 in endothelial cells, thus disrupting tight junctions and increasing blood brain barrier permeability in vitro and in xenograft mice [45]. TLR4 expression and functionality were downregulated in chemoresistant compared to sensitive glioblastoma cells, which resulted in decreased cell viability, proliferation and migration in response to LPS and HMGB1. TLR4 expression was reduced by up to 90% in macrophages when cultured with glioma cells [46].
Breast cancer	The phytochemical tussilagone suppresses triple-negative breast cancer cell progression, invasion and proliferation in association with decreased TLR4 expression and these effects were suppressed by overexpression TLR4 [47]. High expression of TLR4 in breast cancer tissue compared to normal mammary tissue and BC cell lines compared to normal mammary cells. The activation of TLR4 increases breast cancer cell migration [48]. Immunofluorescence assays and analysis of three GEO datasets showed upregulation of TLR4 expression in inflammatory breast carcinoma compared with non-inflammatory breast cancer [49]. Bone marrow-derived progenitors promote lymphangiogenesis by fusing directly with preexisting lymphatic endothelial cells (LECs). This fusion process is dependent on TLR4 pathways. Pharmacological TLR4 inhibition reduces fusion, lymphangiogenesis, lymph node metastasis and tumour spread in vivo [50]. Linoleic acid enhances migration, MMP9 production and invasion of breast cancer cells in vitro, in a TRL4-dependent fashion [51]. Rab27a regulates the secretion of exosomes in breast cancer in a pathway that involves TLR4 to affect angiogenesis and breast cancer (BC) progression [52].
Cervical cancer	TLR4 activation in HeLa cervical cancer cells enhances metastasis to the lymph nodes in nude mice [53]. CRISPR/Cas9 TLR4 knockout significantly reduces cervical cancer cell viability, decreases migration, enhances chemosensitivity, and increases ROS production [54]. Pharmacological activation of TLR4 in cervical cancer cells prior to inoculation to mice increases tumour size, while siRNA TLR4 downregulation has the opposite effect (109). Activation of TLR4 enhances tumour cells proliferation (110). TLR4 activation enhances cervical cancer cell proliferation and stimulates ROS production and expression of HIF-1α [55]. Immunohistochemistry (IHC) demonstrates higher TLR4 expression in invasive cervical cancer compared to cervical intraepithelial neoplasia and no significant difference in TLR4 expression based on tumour stage. The activation of TLR4 induces apoptosis resistance in SiHa cells but not in HeLa cells as they have high and low mRNA and protein levels, respectively [56]. IHC shows downregulation of TLR4 expression with cervical neoplasia progression [57].
Endometrial cancer	The phytochemical bavachinin binds TLR4 in endometrial cancer cells, induces panoptosis and enhances chemosensitivity [58]. Higher TLR4 mRNA expression is associated with better overall survival [35]. The TLR4 gene expression from endometrial cancer has no significant correlation with tumour size, lymph node status, tumour differentiation, or FIGO stage of endometrial cancer [59]. Significant downregulation of TLR4 mRNA expression levels from endometrial tissue biopsies in endometrial adenocarcinoma compared to control postmenopausal women [34].
Head and neck cancer	IHC of oral cancer tissues shows high TLR4 expression is associated with increased cervical lymph node metastasis and poor prognosis. TLR4 activation in human oral carcinoma cells enhances cell migration and invasion [60]. IHC of oral tongue squamous cell carcinoma shows TLR4 expression in primary tumours, local recurrent tumours and metastases. In addition, TLR4 expression seems correlated to invasive potential [61]. TLR4 activation in oropharyngeal squamous cell carcinoma SCC143 cells and mucosal epithelial squamous cell carcinoma SCC78 cells increases cell proliferation without affecting colony formation or migration. TLR4 inhibition decreases proliferation colony formation and migration in all cell lines studied (SCC143, SCC78, FaDu and SCC154. Furthermore, TLR4 activation did not affect cells sensitivity to chemotherapy, while TLR4 inhibition enhances chemosensitivity [62]. LPS activation of TLR4 increased head and neck cancer cell growth and immediate activation of NF-κB and low apoptosis. In comparison, activation by OK-PSA, an immunotherapeutic agent derived from *Streptococcus pyogenes* low-virulence strain used in head and neck cancer, inhibits cancer cell proliferation, and induces late activation of NF-κB and higher apoptosis compared with LPS [63]. The activation of TLR4-NF-κB signalling positively correlates with higher aggressiveness phenotypes in neoplastic laryngeal carcinoma cells [64]. TLR4 activation significantly enhances cell migration in vitro and tumour progression in vivo [65].
Kidney cancer	TLR4 mRNA levels are higher in kidney renal clear cell carcinoma (KIRC) tissue compared to adjacent normal renal tissue, and expression is higher in monocytes/macrophages than other cell types. TRL4 seems protective: close association between TLR4 expression and immune cell infiltration; lower lymph node metastasis; TLR4 knockdown in renal cell adenocarcinoma increases cell proliferation and clonogenicity (without affecting migration); high expression of TLR4 in KIRC correlates with lower tumour grade and stage and improved survival [26]. TLR4 mRNA and protein levels are increased in clear cell renal carcinoma tissues compared to normal tissue. Knockdown of TLR4 in clear cell renal carcinoma cells significantly inhibits cell proliferation, migration and invasion. In addition, high TLR4 expression in tumours is correlated with better overall survival. Suggested mechanism is close association with immune checkpoints and infiltrated immune cells [66]. TLR4 mRNA and protein expression levels is higher in renal cell carcinoma compared to adjacent normal tissue. miR-216a exerts tumour suppressor effects by downregulating TLR4 expression; restoring TLR4 expression in renal cell carcinoma attenuates miR-216a-induced inhibition of proliferation, invasion and migration [67].
Liver cancer	Activation of TLR4-NF-κB signalling significantly decreases liver cancer cell viability [68]. The phytochemical *Polygonatum sibiricum* polysaccharide (PSP) reduced cell proliferation, migration, invasion and EMT of HepG2 and Hep3B cells. LPS reversed these effects, suggesting a pro-cancer effect of TLR4 signalling in this system [69]. TLR4 activation by LPS promotes cell migration and invasion in human liver adenocarcinoma [70].
Lung cancer	TLR4 inhibition significantly reduces non-small-cell lung cancer (NSCLC) cell proliferation and migration and induces apoptosis in vitro. In addition, TLR4 inhibition enhances radiosensitivity both in vitro and in vivo [71]. TLR4 activation (by intratumoral LPS) increases lung tumour size and weight in vivo. In addition, it enhances NSCLC cell proliferation in vitro [72]. TLR4 knockdown significantly inhibited reniformin-induced pyroptosis, migration and invasion in lung cancer cells [73]. The TLR4-active morphine metabolite morphine-3-gluconide upregulates PD-L1 and enhances NSCC-immune escape. Pharmacological TLR4 inhibition prevents this effect [74].
Lymphoma	TLR4 knockout mice exhibits lower mortality rate compared to control mice in radiation-induced thymic lymphoma. In addition, radiation may induce apoptosis and cell injury, via production of DAMPs e.g., HMGB1, which activates TLR4 and promotes carcinogenesis [32].
Melanoma of the skin	TLR4 inhibition reduces melanoma cell viability, migration, invasion and colony formation, and enhances cell apoptosis [75].
Myeloma	TLR4 participates in bortezomib-induced drug resistance in myeloma cells [76,77]. Decreased proliferation and apoptosis induction of multiple myeloma cells by the phytochemical andrographolide were increased by TLR4 siRNA knockdown [78].
Oesophageal cancer	TLR4 activation enhances oesophageal cancer cell proliferation, migration in vitro. In addition, intratumoural LPS administration increases tumour volume in vivo [65]. Activation of TLR4-NF-κB in oesophageal squamous cell carcinoma promotes cell invasion and metastasis [79,80]. IHC of oesophageal squamous cell carcinoma (ESCC) tissue samples shows that TLR4 protein expression from the lowest expression to the highest: normal tissue, low grade intraepithelial neoplasia, high grade intraepithelial neoplasia and early stage ESCC, which later decreased in the advanced ESCC stage. In addition, high TLR4 protein expression is an independent prognostic factor for better overall survival [81]. TLR4 knockdown in oesophageal cancer cells induces apoptosis and decreases cell proliferation [82].
Ovarian cancer	Cox regression analysis of IHC performed on a tissue microarray (TMA) of stage III-IV high-grade serous ovarian cancer shows that high TLR4 expression is associated with poorer progression-free survival (PFS) [83]. TLR4 activation in ovarian cancer cells induces cell proliferation and invasion. In addition, TLR4 inhibition enhances chemosensitivity and induces apoptosis in ovarian cancer cells [84,85]. TLR4 plays a pivotal role in taxol resistance in ovarian cancer; TLR4 overexpression activates PI3K/AKT signalling, upregulates the expression of the androgen receptor and activates IL-6 signalling. TLR4 knockdown downregulates androgen receptors in Taxol-resistant ovarian cancer cells [86]. Paclitaxel induces TLR4–NF-κB activation, which facilitates calreticulin (CALR) translocation to the cancer cell surface, thereby potentiating immunogenic cell death (ICD) in ovarian cancer cells [87]. TLR4 inhibition exhibits synergistic anti-tumour effect with chemotherapy in both ovarian and breast cancer cells [88].
Pancreatic cancer	Inhibiting TLR4 enhances pancreatic cancer cells sensitivity to chemotherapy [89]. TLR4 mRNA and protein expression levels are higher in pancreatic cancer than in normal pancreatic cells. In addition, TLR4 inhibition suppresses cancer cells migration [90]. The soluble immune checkpoint sB7-H3 promotes pancreatic cancer invasion and metastasis via TLR4; shRNA TLR4 silencing reduces sB7-H3-induced metastasis of pancreatic cancer cells in mice [91]. TLR4 inhibition by *Lactobacillus casei* and *Lactobacillus reuteri* decreases TLR4 and MyD88 mRNA expressions and decreases cell viability in pancreatic cell lines. TLR4 activation enhances cell migration and invasion. In addition, pretreatment of pancreatic cancer cells with *Lactobacillus casei* and *Lactobacillus reuteri* attenuates M2 macrophage polarisation [92].
Prostate cancer	TLR4-NF-κB signalling pathway activation via S100A9 promotes prostate cancer cell invasion [93]. Higher TLR4 mRNA levels in prostate cancer tissue compared to adjacent tissue [94]. TLR4 activation by LPS decreases prostate cancer cell proliferation [95].
Stomach cancer	LPS activation of TLR4 promotes gastric cancer proliferation and invasion [96,97]. TLR4 activation by LPS enhances gastric cancer cell proliferation in vitro [98].

**Table 2:** TLR4 agonists used in clinical trials for cancer treatment (from https://clinicaltrials.gov, accessed on 18 December 2025).

NCT Number	Conditions	Interventions	Sponsor	Outcome
NCT02180698	Stage III and IV Adult Soft Tissue Sarcoma	Combined treatment of radiation and synthetic TLR4 agonist glucopyranosyl lipid A-stable-emulsion (GLA-SE) for treating unresectable and metastatic soft tissue sarcoma.	Fred Hutchinson Cancer Center	Simultaneous radiotherapy and intratumoural GLA-SE enhance durable local control of metastatic sarcoma, and the expansion of intratumoural T-cells. The study has low number of patients as a limitation to evaluate the clinical efficacy with no placebo control [119].
NCT02320305	Stage IIA IIB IIC IIIA IIIB IIIC and IV Skin Melanoma	Combined treatment of melanoma antigen recognised by T-cells 1 (MART-1) antigen (peptide vaccine) with GLA-SE or SE alone as control for treating stage II-IV resected melanoma.	Mayo Clinic	The use of GLA-SE with vaccine is well-tolerated. No significant difference between GLA-SE and SE in antigen-specific immune response. However, higher proinflammatory cytokines were produced in SE compared to GLA-SE group [120].
NCT02015416	Melanoma, Ovarian Cancer, Sarcoma, Non-small Cell Lung Cancer, Breast Cancer	Combined treatment of recombinant NY-ESO-1 antigen and GLA-SE for treatment of melanoma, ovarian cancer, sarcoma, NSCLC, and breast cancer.	Immune Design, a subsidiary of Merck & Co., Inc. (Rahway, NJ, USA)	Not available yet
NCT02035657	Merkel Cell Carcinoma	Synthetic intratumoural administration of TLR4 agonist Glucopyranosyl Lipid A—Stable Emulsion (GLA-SE).	Immune Design, a subsidiary of Merck & Co., Inc. (Rahway, NJ, USA)	Not available yet
NCT02406781	Sarcoma	Combined treatment pembrolizumab, TLR4 agonist G100 (synthetic lipid A derivative) intratumoural injection, and cyclophosphamide in patients with advanced pretreated soft tissue sarcoma.	Institut Bergonie	Limited clinical activity when combined with PD1 inhibitor, with no clear association between tumour shrinkage and the increased inflammatory status after TLR4 activation via G100. Furthermore, the first endpoint of the study (6-month non-progression rate was not reached) [121].
NCT03447314	Neoplasms	Combined treatment of GSK1795091 (synthetic lipid A analogue TLR agonist) and Immunotherapies (anti-OX40 monoclonal antibody, anti-ICOS monoclonal antibody or pemrolizumab) in advanced solid tumour subjects.	GlaxoSmithKline	Conclusions regarding anti-tumour effect of TLR4 agonist cannot be reached as the manufacturing formula of the TLR4 agonist was modified during the trial [122].
NCT02798978	Healthy volunteers	GSK1795091 (TLR4 agonist) vs. placebo in healthy volunteers. A phase 1 two-parts study, part 1: dose escalation, part 2: a parallel group.	GlaxoSmithKline	IV administration of TLR4 agonist is well tolerated in healthy volunteers with no serious adverse events, favourable pharmacokinetics and dose-dependent stimulatory effect of immune cell [123].

**Table 3:** Role of gelatinases in cancer.

Cancer Type	Gelatinase Observed Effect
Bladder cancer	Higher serum MMP9 levels in bladder cancer patients compared to healthy controls, with higher serum levels of MMP9 in T-stage bladder cancer compared to N- or M-stages [155]. MMP9 CRISPR/Cas9 knockout in T24 bladder cancer cells inhibits cell proliferation, invasion and migration in vitro [156]. Chemotherapeutic treatments gemcitabine and cisplatin alone and in combination differentially affect MMP2 and MMP9 gene expression in bladder cancer cell lines, with no overall trend available [157]. Bioinformatic analysis of bladder cancer samples in TCGA database shows that lower MMP9 RNA levels correlate with higher survival rate, and the expression of MMP9 RNA levels is increased in tumour samples compared to normal samples. In addition, MMP9 was positively correlated to M0 macrophages in the TME and negatively correlated to activated dendritic cells or monocytes [158]. Radioresistant bladder cancer cells show higher mRNA levels of both MMP2 and MMP9 compared to their parental cells. siRNA downregulation of COX-2 significantly downregulates gelatinases mRNA levels in bladder cancer cells [159]. MMP9 activity increases in high grade tumour tissue, while MMP2 activity increases in low grade tumour tissue. MMP9 increase is proportionally higher than that of MMP2 [160]. Higher protein levels of MMP2 in human bladder cancer tissue compared to normal tissue. MMP-2 levels are associated with the clinical disease stage and poor survival rate [161]. MMP2 and MMP9 were significantly elevated in blood and urine samples from malignant compared to benign and normal groups. MMP-2/TIMP-2 and MMP-9/TIMP-2 can discriminate high-grade from low-grade and advanced from early-stage tumours [162]. Meta-analysis of 23 case-control studies shows that MMP9 protein levels (IHC or ELISA) upregulation in bladder cancer compared to healthy tissues. MMP9 also increases with tumour grade [163]. Low MMP2 protein serum expression levels is associated with increased invasiveness and predicts lymph node metastasis in urethral carcinoma of the bladder [164]. Upregulation of both MMP9 and TIMP2 mRNA levels in superficial bladder cancer tissue of patients with tumour recurrence compared to patients without recurrence with no significant difference in MMP2 mRNA levels. Significantly lower recurrence-free survival of patients with elevated MMP-9 or TIMP-2 mRNA compared to patients with normal MMP-9 or TIMP-2 expression [165]. Low serum proMMP2 levels or low serum TIMP-2 levels are associated with poor prognosis of bladder cancer patients [166]. A meta-analysis of case-control studies evaluating MMP polymorphisms and susceptibility to bladder cancer shows that MMP-2-1306 C/T polymorphism (which controls MMP2 expression) is associated with bladder cancer risk [167]. Bladder cancer patients have higher serum levels of MMP9, TIMP-2, and MMP9/TIMP-2 ratio than healthy controls, with no differences in MMP2 serum levels or MMP2/TIMP2 ratio [168]. RT-PCR of malignant bladder cancer tissue shows higher MMP9 mRNA levels and lower MMP2, TIMP-1, and TIMP-2 compared to normal bladder tissue. In addition, MMP9 mRNA levels are higher levels in invasive tumours compared to superficial tumours [169]. IHC of tissue microarray of lymph-node positive bladder cancer shows increasing MMP9 expression from the lowest to the highest: normal urothelium, primary tumour, lymph-node metastasis. MMP2 increases from normal urothelium to primary tumour but expression then decreases in lymph-node metastases. Neither MMP2 nor MMP9 increased at tumour invasion front compared to centre and the invasive front [170].
Colorectal cancer	MMP2 inhibition decreased SW620 cells migration and invasion in the chorioallantoic membrane assay [171]. MMP9 activity in colon cancer tissue is higher than in tumour-adjacent tissue followed by distant healthy colon tissue. In addition, MMP9 content was the highest in stage II of colon cancer compared to other stages compared to stages I III or IV [172]. Serum protein levels of both MMP2 and MMP9 are significantly higher in colorectal adenocarcinoma patients than in healthy controls. In addition, serum protein level of both gelatinases is higher in lymphovascular invasion positive patients compared to lymphovascular invasion negative patients. RT-PCR shows higher gene expression of MMP2 and MMP9 in colorectal cancer tissue compared to normal tissue. MMP9 expression is higher in stage IV tissue compared to stage II, and stage III colorectal cancer tissue [173]. A meta-analysis of colorectal cancer tissue shows that high MMP2 protein expression in tumour tissue is associated with significantly decreased overall survival and poorer disease-free survival in colorectal cancer patients. No significant correlation between MMP9 protein in tumour tissue and overall survival or disease-free survival was found [174]. IHC staining of tumour tissue and adjacent normal tissue shows that high MMP2 protein expression is associated with advanced stage of colorectal cancer, metastasis, vascular invasion, perineural infiltration and lymph-node involvement. In addition, in vitro inhibition of MMP2 significantly reduces colorectal cancer cell proliferation, migration and invasion and induces apoptosis [175]. IHC staining of colorectal cancer tissue and adjacent healthy tissue shows significant upregulation of MMP9 protein levels in colorectal cancer tissue compared to adjacent healthy tissue. In addition, MMP9 levels in tumour tissue are associated with higher degree of tumour differentiation, increased depth of invasion, metastasised lymph-node, increased tumour size, and distant metastasis [176]. IHC of CRC tissue shows higher MMP9 protein levels associated with higher cancer stage [177]. Significant increase in serum MMP9 levels of colorectal cancer patients compared to healthy controls. MMP9 proposed as diagnostic biomarker. In addition, serum MMP9 levels are positively associated with serum miR-26a and serum miR-26b levels [178]. Normal colon epithelial cells show lower MMP2, and MMP9 protein expression levels compared to human colorectal cancer cells [179]. Serum MMP9 protein expression levels are significantly upregulated in colorectal patients compared to healthy controls with no significant diagnostic value [180]. Significant increase in MMP9, TIMP-1, TIMP-2 serum antigen concentrations but not MMP2 in colorectal cancer patients compared to healthy controls or colorectal adenoma. MMP9, TIMP-1 and TIMP-2 serum protein levels are upregulated in Dukes advanced tumour stages (C + D) compared to earlier stages (A + B). Furthermore, neither MMP2 nor MMP9 serum protein levels are significantly different in patients with or without liver metastases [181]. Upregulated MMP9 protein expression levels and downregulated TIMP-2 protein expression levels in colorectal cancer tissue compared to normal adjacent healthy tissue. In addition, MMP9 significantly increased with the depth of tumour invasion, increased lymph-node metastasis, increased distant metastasis, and TNM stage. High MMP-9 expression correlates with the poor survival. MMP9 knockdown significantly reduces colorectal cancer cell migration, invasion and angiogenesis in vitro, while MMP9 expression increases cell migration, invasion and angiogenesis [182]. A meta-analysis of 12 publications assessing the clinical value of MMP9 as a biomarker shows elevated serum MMP9 levels in colorectal cancer and significant association with colorectal cancer risk. It also concludes that serum MMP9 levels in colorectal cancer patients has moderate diagnostic accuracy for colorectal cancer [183]. Serum MMP9 protein levels increased colorectal cancer stage. In addition, higher serum MMP9 levels were found in patients with pericolonic lymph-node involvement, or metastatic colorectal cancer compared to no involvement of lymph-nodes and non-metastatic colorectal cancer serum levels, respectively [184]. IHC staining showed MMP-9 positivity associated with low WHO grade. In Dukes’ B tumours, MMP-9 negativity associated with poor survival, and MMP-9 positivity acted as an independent prognostic marker [185].
Brain cancer	Inhibition of MMP9 via selective engineered N-TIMP2 variant significantly inhibits glioblastoma (GBM) cell proliferation, spheroid spread, invasion and colony formation [186]. Higher MMP9 expression in GBM tissue than in non-cancerous brain tissue. In addition, higher levels of MMP9 expression are associated with poor overall survival [187]. Temozolomide decreases GBM cell viability in vitro and upregulates MMP9 without changing MMP2 expression. Furthermore, in a mouse model of subcutaneous implantation of glioblastoma, WB of tumour shows higher MMP9 protein levels in the temozolomide-group compared to the DMSO group. Interestingly, after TMZ treatment, MMP2 activity also increased in tumour tissue in vivo [188]. Electrical field stimulation enhances U87 GBM cell migration and MMP9 protein but not MMP2 [189]. Combined with dynamic susceptibility contrast- perfusion weighted imaging (DSC-PWI), increased serum levels of MMP2 and MMP9 allow to distinguish recurrence from non -recurrence after postoperative radiotherapy of GBM [190]. Treatment of endothelial cells by glioblastoma-conditioned media enhances endothelial cell migration and upregulates mRNA and protein levels of MMP9 compared to untreated endothelial cells [191]. Temozolomide causes dose- and time-dependent downregulation of MMP2 and MMP9 mRNA and activity levels [192]. The phytochemical amentoflavone significantly reduces MMP2 and MMP9 in GBM by decreasing NF-κB—also reducing glioblastoma cell growth, migration and invasion [193].
Breast cancer	IHC staining of primary breast tumour tissue shows that MMP2 protein levels are significantly correlated with BC stage but not grade. MMP2 overexpression is associated with poorer prognosis [142]. Higher MMP2 and MMP9 activities in serum of breast carcinoma patients compared to healthy controls. Furthermore, a positive correlation between gelatinases levels and c-erb-2 overexpression (linked to aggressive phenotypes), and inverse correlation with oestrogen receptor expression [194]. IHC of breast cancer tissue shows detectable levels of MMP9 in cancer cells followed by stromal cells, and undetectable levels in normal breast tissue. Low MMP9 levels are associated with more aggressive phenotypes, such as larger tumour volume, progesterone receptor negative or strong positive tumours, and stage III–IV disease. In addition, node-negative breast cancer with MMP9-positive tumour tissue has low risk of relapse and death, making MMP9 expression a prognostic factor in node-negative breast cancer [195]. Triple-negative breast cancer cells exhibit higher levels of MMP2 and MMP9 mRNA and protein levels compared to hormone-responsive breast cancer cells. In addition, MMP9 knockdown or its pharmacological inhibition in breast cancer cells suppresses cell invasion and pharmacological inhibition of MMP9 in xenograft model suppresses metastases to lung and liver, with decreasing tumour weight in vivo [196]. Protein levels of MMP9 in breast tumour tissue are higher than normal breast tissue. In addition, TNF-α enhances MMP9 mRNA and protein levels with enhanced breast cancer cell invasion, meanwhile MAPK(ERK, JNK, p38) or NF-κB inhibitors suppress TNF-α-induced MMP9 protein levels upregulation [197]. Gold nanoparticles suppress angiogenesis in association with decreased protein levels of MMP9, NF-κB, mTOR and PDL-1 expression in breast cancer cells [198]. Elevated MMP2 and MMP serum protein level in breast cancer patients compared to healthy controls regardless of metastasis status. Elevated serum MMP9 but not MMP2 levels are associated with increased tumour size and poor prognosis [199]. MMP9 inhibition significantly suppresses cell invasion and proliferation in breast cancer cells [200]. The phytochemical dihydroartemisinin suppresses breast cancer-induced neovascularisation in the CAM model in parallel with MMP2 and MMP9 protein levels and activities [201]. The phytochemical gypensapogenin H significantly inhibits breast tumour cell migration and invasion, with decreased MMP9 protein levels both in vivo and in vitro [202]. The serum protein levels of MMP9 are significantly upregulated in breast cancer patients at diagnosis and after chemotherapy compared to healthy controls. MMP-2 and MMP-9 serum levels negatively correlated with tumour size and metastatic lymph nodes after chemotherapy [203]. Higher MMP9 protein levels in breast cancer cells compared to non-tumorigenic cells in vitro. MMP9 siRNA decreases breast cancer cell proliferation, migration and invasion [204]. Higher serum MMP9 and TIMP-1 protein levels of breast cancer patients before and after treatment in comparison with healthy controls (treatment groups: (a) radiotherapy followed by chemotherapy, or (b) chemotherapy followed by radiotherapy, or (c) concomitant radio chemotherapy). In addition, while all treatment groups reduce MMP9 and TIMP-1 serum levels, groups c has more pronounced reduction effect compared to group a and group b [205]. Higher mRNA and protein levels of MMP2 and MMP9 in breast cancer cells compared to normal breast cells. IHC shows higher MMP2 and MMP9 protein levels in breast cancer tissue compared to adjacent normal tissue. Furthermore, increased MMP2 and MMP9 protein levels in IHC of breast cancer tissue was correlated with increased lymph-node metastasis occurrence and tumour staging but not tumour size, whereas lower MMP2 and MMP9 protein levels are indicating better prognosis [206].
Cervical cancer	The natural polyphenol bergenin reduces cervical cancer cell viability, migration and angiogenesis in association with decreased MMP9 protein levels [207]. A synthetic curcumin analogue significantly reduces cells migration and invasion in parallel with suppressing MMP9 mRNA, protein and activity [208]. IHC of cervical cancer tissue shows that MMP9 protein expression is positively associated with lymph-node metastasis expression [209]. Significant upregulation of MMP9 mRNA levels in cervical cancer tissue compared to healthy cervical tissue, with significant co-expression of MMP9 and VEGF in cervical cancer tissue [210].
Endometrial cancer	A meta-analysis study shows MMP9 protein overexpression in endometrial cancer tissue is positively associated with tumour grade, lymph node metastasis and increased risk of endometrial cancer [211]. IHC shows nearly undetectable MMP2 and MMP9 protein levels in normal endometrium, endometrial polyps and endometrial cancer tissue [212]. IHC shows that MMP2 and MMP9 overexpression is associated with poor survival rate. In addition, MMP9 protein levels correlate with endometrial cancer clinical stage while MMP2 protein levels do not [213].
Head and neck cancer	IHC and RT-PCR show significant upregulation of both MMP2 and MMP9 mRNA and protein levels in laryngeal cancer tissue compared to adjacent healthy tissue controls. In addition, MMP2 and MMP9 high expressions are correlated with poor survival outcome and advance TNM stage [214]. Photodynamic therapy combined with carboplatin treatments significantly reduces MMP2 and MMP9 protein levels in laryngeal cancer cells together with viability, migration and invasion [215]. S100A4 increases Hep-2 cell invasion via MMP9 upregulation of MMP9 as demonstrated by MMP9 siRNA [216]. SOX-2 increases laryngeal cancer cell invasion and migration via upregulation of MMP2 as demonstrated by MMP2 siRNA and pharmacological inhibition [217]. MMP9 down regulation inhibits laryngeal squamous cell carcinoma cells cell viability, and invasion in vitro. In addition, intratumoural MMP9 shRNA decreases tumour weight in vivo [218].
Kidney cancer	Decreased ACHN renal adenocarcinoma cell migration and viability in parallel with decreased MMP2 and MMP9 activities after treatment by curcuminoid alone or combined with TNF-related apoptosis-inducing ligand (TRAIL) in vitro [219]. Knockdown of metastatic-associated protein 2 (MTA2) reduces MMP9 activity, mRNA and protein levels without affecting MMP2 activity in renal cancer cells in vitro [220]. Gelatinases increase with tumour grade in renal cancer [221]. The Chinese medicine *Eupatorium fortunei* induces suppression of metastasis and angiogenesis in HT1080 cells in parallel with inhibition of PMA-induced MMP9 mRNA upregulation [222].
Liver cancer	Radiation enhances invasion of three hepatocellular carcinoma cell lines but not normal hepatocellular cells in vitro. Radiation upregulates MMP9 mRNA, protein and activity. Furthermore, antisense MMP9 oligonucleotide significantly suppresses radiotherapy-induced cell invasion in both in vitro and in vivo [223]. In a rat model of diethylnitrosamine-induced hepatocellular carcinoma, cyclophosphamide metronomic therapy significantly suppressed MMP2 and MMP9 activities as well as metastasis and improved survival—and the effect was more pronounced than maximum tolerated dose therapy [224]. Nanofibres made of doxorubicin conjugated with an hexapeptide MMP inhibitor significantly inhibits MMP2 and MMP9 activities, HCC metastasis, decreases tumour volume and tumour wight with enhancing survival rate compared to doxorubicin alone or MMP inhibitor alone in HCC xenograft-bearing mice [225]. MicroRNA-26b (which is decreased in liver cancer tissue compared to adjacent normal tissue) lowers MMP9 protein levels in HCC compared to negative control and increases apoptosis levels [226]. Recombinant human sonic hedgehog (SHH) N-terminal peptide enhances cell adhesion, migration and invasion, and upregulates MMP2 and MMP9 mRNA, protein and activities in vitro in HCC. On the other hand, anti-SHH decreases both MMP2 and MMP9 mRNA levels and activities in HCC cells [227].
Lung cancer	The synthetic MMP9 inhibitor (MG-3G) exhibits selective cell proliferation inhibition of NSCLC cells harbouring T790M mutation in vitro and directly binds to MMP9 protein and stabilises it in the cellular microenvironment. Additionally, MG-3G attenuates T790M-mutant NSCLC cell invasion and migration and induces apoptosis. Furthermore, it decreases protein expression of MMP2, MMP9, vimentin, E-cadherin and N-cadherin, thus decreasing EMT in vitro [228]. Serum levels of MMP9 but not MMP2 are significantly elevated in lung cancer compared to healthy donors [229]. *Streptococcus pneumoniae* presence in lung cancer tissue correlates with advanced tumour stage, brain metastases, reduced overall survival and MMP9 but not MMP2 protein levels in tumours [230]. siRNA MMP2 downregulation increases apoptosis and reduces lung cancer cell invasion migration, and angiogenesis in vitro. In a metastatic lung tumour model, treatment of existing tumours by MMP2 siRNA adenovirus decreased tumour growth and metastasis [231,232]. Radiation enhances lung cancer cell invasion, and increases MMP9 mRNA and protein levels, while MMP9 siRNA significantly suppresses the induced invasion in vitro. In addition, radiation enhances pulmonary metastasis, which is significantly reduced by either MMP9 or both MMP2 and MMP9 knockdown in vivo. Furthermore, MMP9 inhibitor reduces radiation-induced pulmonary metastasis [233]. Higher MMP9 protein expression in lung cancer cells (detected by western blot) and lung cancer tissue (detected by IHC and western blot) compared to normal lung cells and normal lung tissue, respectively. MMP9 silencing enhances radiation-induced decrease in cell proliferation, migration and invasion. Furthermore, it enhances radiation-induced lung cancer cell apoptosis in vitro [234]. MMP2-735C/T and MMP9-1562C/T polymorphisms are associated with increased risk of lung cancer [235]. Serum levels of MMP9 are significantly elevated in lung cancer patients compared to healthy control [236]. miR-21 treatment of lung cancer cells activates AKT/Caspase 3/MMP2 and MMP9 signalling and enhances cells migration and invasion in vitro [237]. Radioresistant A549 (A549-IR) cells exhibits enhanced migration and invasion, with MMP2 mRNA and protein upregulation, whereas MMP9 has low gene expression and high protein expression levels compared to parenteral A549 cells. Both gelatinase activities are higher than parental control [238].
Lymphoma	Active primary central nervous system lymphoma patients have higher serum MMP9 levels compared to patients without radiographic disease [239]. Glutamate receptor activation significantly increases MMP9 production by lymphoma cells in vitro and their engraftment in vivo [240]. ICH of lymphoma tissue shows no correlation between MMP9 protein levels and tumour stage, type or prognosis in children lymphoma [241].
Melanoma of the skin	Curcumin derivative inhibits MMP2 and MMP9 activities in gelatin zymography together with inhibition of melanoma cell proliferation, migration and invasion in vitro [242]. The phytochemical galbanic acid inhibits MMP2 and MMP9 production in melanoma cells with suppression of cell migration and invasion [243]. In vitro treatment of melanoma cells with LPS does not change MMP2 activity but increases MMP9 activity at concentrations up to 25 µg/mL followed by decreased activity. Additionally, both PMA and TNF-α do not change MMP2 activity but upregulates MMP9 activity [244]. Higher circulating MMP9 protein levels in melanoma patients’ plasma compared to healthy controls plasma. Furthermore, higher MMP9 activity from BRAF v600E mutation melanoma plasma sample compared to wild type melanoma plasma MMP9 activity. Higher serum MMP9 protein levels and activity are associated with lymph node involvement, ulceration and negative prognostic. Peripheral blood monocytes from the melanoma group treated with osteopontin shows higher MMP9 protein levels relative to control group. Besides, NF-κB inhibitor treatment of PBMCs decreases MMP9 protein levels in melanoma group without significant change in control group [245].
Myeloma	Bone marrow plasma and culture supernatants have higher MMP2, MMP9, TIMP1 and TIMP2 median protein levels compared to healthy controls. However, no correlation between protein levels of gelatinases, TIMP1 or TIMP2 and the clinical stage [246]. The in vitro transendothelial invasion of human CD138^+^ multiple myeloma cells derived from bone marrow of multiple myeloma patients is mediated by increased production of MMP9; MMP9 antibody inhibits transendothelial invasion and endothelial cells upregulate multiple myeloma MMP9 production [247]. Resveratrol inhibits multiple myeloma cell proliferation, invasion, MMP2 and MMP9 activities and protein levels [248].
Oesophageal cancer	TCGA database shows overexpression of MMP9 mRNA levels in oesophageal squamous cell carcinoma (ESCC) tissue, but not oesophageal adenocarcinoma (EAC) tissue compared to healthy normal tissue. Furthermore, MMP9 mRNA level is associated with oesophageal cancer stage. GEP microarray database shows upregulation of MMP9 mRNA levels in invasive oesophageal squamous cancer cell cells compared to non-invasive group. IHC shows higher protein levels of MMP9 in ESCC tissue compared to adjacent normal tissue in Kazakh patients. Higher MMP9 protein level is associated with depth of invasion, lymph node metastasis and poor prognosis [249,250]. Higher serum MMP9 protein levels in oesophageal cancer patients compared to healthy group. Serum MMP9 increases with higher oesophageal cancer stage and increased tumour size >4 cm. However, MMP9 protein levels are not dependent on the tumour invasion depth. Furthermore, MMP9 serum protein levels have less diagnostic specificity than serum protein levels of tumour markers (SCC-Ag-93 and CEA) [251].
Ovarian cancer	In vitro Neuropeptide FF (NPFF) increases ovarian cancer cell invasion without affecting cell proliferation compared to untreated control. N parallel, NPFF treatment of ovarian cancer cells upregulates MMP9 mRNA and protein levels in a time-dependent response up to 12 h then the MMP9 mRNA levels decreases. No effect on MMP2 mRNA levels [252]. Higher MMP2 mRNA level is associated with better ovarian cancer response to chemotherapy [253]. Oxytocin treatment of ovarian cancer cells significantly and dose-dependently inhibits MMP2 protein expression and activity and suppress cells metastasis in vitro [254]. Environmental pollutant perfluorooctanoic acid (PFOA) enhances ovarian cancer cell invasion and migration and induces upregulation of the gelatinase’s mRNA and protein in a NF-κB dependant fashion [255].
Pancreatic cancer	Knocking down stress-induced phosphoprotein 1 (STIP1) significantly inhibits pancreatic cancer cells invasion and downregulates MMP2 and MMP9 mRNA and protein levels [256]. Ablation of MMP9 in the host, but not in the cancer cells, increases pancreatic cancer aggressiveness: in the KPC spontaneous pancreatic cancer mouse model, MMP9 gene disrupted mice exhibit larger, more invasive pancreatic adenocarcinomas with more stroma compared to MMP9-expressing mice. Yet, knockdown of MMP-9 in pancreatic cancer cell lines did not induce invasive growth but rather reduced proliferation, migration and invasion in vitro [257]. COX-2 inhibition increases invasion and MMP2 protein levels in pancreatic cancer cells in vitro [258]. The upregulation of MMP9 protein levels in pancreatic cancer tissue vascular invasion, lymph node invasion, liver metastases and TNM stage [259].
Prostate cancer	Pterocarpanquinone LQB-118 inhibits prostate cancer cell migration, and invasion. In addition, it decreases MMP9 mRNA levels compared to untreated prostate cancer cells [260]. MMP2 mRNA expression levels in atypical small acinar proliferation (precursor lesion of prostate cancer) significantly associated with prostate cancer risk [261]. COX-2 inhibition suppresses prostate cancer invasion and proliferation in vitro. It also downregulates MMP2 and MMP9 protein levels [262].
Stomach cancer	MMP2 and MMP9 activities are increased in regional metastatic tissue of gastric cancer but MMP2 activity significantly decreases in distant metastasis [263]. HER2 knockdown decreases gastric cancer cell migration and invasion. Additionally, it decreases MMP9 protein levels without affecting MMP2 protein levels [264]. MMP9 protein level in gastric cancer tissue is not correlated to overall survival but MMP2 protein level is an independent prognostic factor [265].

## Data Availability

No new data was created. Not applicable.

## Abbreviations

The following abbreviations are used in this manuscript:

TLR4	Toll-like receptor 4
ECM	Extracellular matrix
MMP	Matrix metalloproteinase
TIMPs	Tissue inhibitors of metalloproteinases
DAMPs	Damage-associated molecular patterns
PAMPs	Pathogen-associated molecular pattern.
LPS	Lipopolysaccharide
LBP	LPS-binding protein
TIR	Toll/interleukin-1-receptor
TIRAP	TIR domain-containing adaptor protein
MyD88	Myeloid differentiation primary response protein 88
IL	Interleukin
TRIF	TIR domain-containing adaptor inducing interferon-β
TRAM	TRIF-related adaptor molecule
NF-κB	Nuclear factor kappa b
TNF-α	Tumor necrosis factor alpha
iNOS	Inducible nitric oxide synthase
IFN-γ	Interferon gamma
PD-L1	Programmed death-ligand 1.
CAR-T	Chimeric antigen receptor t cell therapy.
HMGB1	High mobility group box 1.
ROS	Reactive oxygen species.
CRISPR-Cas9	Clustered regularly interspaced short palindromic repeats and CRISPR-associated protein 9
IHC	Immunohistochemistry
FIGO	International federation of gynecology and obstetrics
KIRC	Kidney renal clear cell carcinoma
ESCC	Osophageal squamous cell carcinoma
PFS	Progression-free survival
TMA	Tissue microarray
CALR	Calreticulin
ICD	Immunogenic cell death
GLA-SE	Glucopyranosyl lipid a in stable emulsion
MART-1	Melanoma Antigen Recognied byT cells 1
NSCLC	Non–small cell lung cancer
PD-1	Programmed cell death protein 1
TCGA	The cancer genome atlas
ELISA	Enzyme-linked immunosorbent assay
GBM	Glioblastoma
DMSO	Dimethyl sulfoxide
WB	White blood
CAM	Chorioallantoic membrane
RT-PCR	Reverse transcription polymerase chain reaction
PMA	Phorbol 12-myristate 13-acetate
ESCC	Esophageal squamous cell carcinoma
NPFF	Neuropeptide ff
STIP1	Stress-induced phosphoprotein 1
PFOA	Pollutant perfluorooctanoic acid
WHO	World health organization
siRNA	Small interfering RNA
TOPK	T-LAK cell-originated protein kinase
SNP	Single nucleotide polymorphism
HCC	Hepatocellular carcinoma
RAGE	Receptor for advanced glycation end products
